# Planned Repeat Cesarean Section at Term and Adverse Childhood Health Outcomes: A Record-Linkage Study

**DOI:** 10.1371/journal.pmed.1001973

**Published:** 2016-03-15

**Authors:** Mairead Black, Siladitya Bhattacharya, Sam Philip, Jane E. Norman, David J. McLernon

**Affiliations:** 1 Division of Applied Health Sciences, University of Aberdeen, Aberdeen, United Kingdom; 2 Diabetes Research Unit, NHS Grampian, Aberdeen, United Kingdom; 3 Tommy’s Centre for Maternal and Fetal Health, University of Edinburgh, Edinburgh, United Kingdom; 4 Medical Statistics Team, Division of Applied Health Sciences, University of Aberdeen, Aberdeen, United Kingdom; King’s College London, UNITED KINGDOM

## Abstract

**Background:**

Global cesarean section (CS) rates range from 1% to 52%, with a previous CS being the commonest indication. Labour following a previous CS carries risk of scar rupture, with potential for offspring hypoxic brain injury, leading to high rates of repeat elective CS. However, the effect of delivery by CS on long-term outcomes in children is unclear. Increasing evidence suggests that in avoiding exposure to maternal bowel flora during labour or vaginal birth, offspring delivered by CS may be adversely affected in terms of energy uptake from the gut and immune development, increasing obesity and asthma risks, respectively. This study aimed to address the evidence gap on long-term childhood outcomes following repeat CS by comparing adverse childhood health outcomes after (1) planned repeat CS and (2) unscheduled repeat CS with those that follow vaginal birth after CS (VBAC).

**Methods and Findings:**

A data-linkage cohort study was performed. All second-born, term, singleton offspring delivered between 1 January 1993 and 31 December 2007 in Scotland, UK, to women with a history of CS (*n* = 40,145) were followed up until 31 January 2015. Outcomes assessed included obesity at age 5 y, hospitalisation with asthma, learning disability, cerebral palsy, and death. Cox regression and binary logistic regression were used as appropriate to compare outcomes following planned repeat CS (*n* = 17,919) and unscheduled repeat CS (*n* = 8,847) with those following VBAC (*n* = 13,379).

Risk of hospitalisation with asthma was greater following both unscheduled repeat CS (3.7% versus 3.3%, adjusted hazard ratio [HR] 1.18, 95% CI 1.05–1.33) and planned repeat CS (3.6% versus 3.3%, adjusted HR 1.24, 95% CI 1.09–1.42) compared with VBAC. Learning disability and death were more common following unscheduled repeat CS compared with VBAC (3.7% versus 2.3%, adjusted odds ratio 1.64, 95% CI 1.17–2.29, and 0.5% versus 0.4%, adjusted HR 1.50, 95% CI 1.00–2.25, respectively). Risk of obesity at age 5 y and risk of cerebral palsy were similar between planned repeat CS or unscheduled repeat CS and VBAC. Study limitations include the risk that women undergoing an unscheduled CS had intended to have a planned CS, and lack of data on indication for CS, which may confound the findings.

**Conclusions:**

Birth by repeat CS, whether planned or unscheduled, was associated with an increased risk of hospitalisation with asthma but no difference in risk of obesity at age 5 y. Greater risk of death and learning disability following unscheduled repeat CS compared to VBAC may reflect complications during labour. Further research, including meta-analyses of studies of rarer outcomes (e.g., cerebral palsy), are needed to confirm whether such risks are similar between delivery groups.

## Editors' Summary

**Background:**

Women who have had a previous cesarean section (CS) face a slightly elevated risk of negative outcomes during a subsequent vaginal birth. This is because, in rare cases, the abdominal CS scar ruptures during labor, which may cause complications and serious problems, such as oxygen deprivation to the brain of a child. As a result, pregnant women who have had a previous CS and their doctors sometimes make a decision to plan a repeat CS instead of a vaginal birth after cesarean section (VBAC). Besides vaginal birth being “more natural,” some studies have suggested that exposure to the mother’s microbiome (the bacteria and fungi that live in the vagina and gut) in the birth canal might be important for the healthy development of the infant’s own microbiome and its immune system. Consistent with this notion, some studies have reported higher levels of allergies (including eczema and asthma) and overweight or obesity in children born by CS.

**Why Was This Study Done?:**

Scotland keeps detailed records of births and childhood health data, which allow researchers to study possible links between type of birth and subsequent childhood health outcomes. In this study, the researchers focused on singleton births (as opposed to twins, triplets, etc.) to mothers who have had a previous CS. In this group, where the decision about a preferred delivery mode is likely made before labor starts, they examined how childhood health outcomes compared between repeat CS and VBAC.

**What Did the Researchers Do and Find?:**

The researchers studied all second births between 1 January 1993 and 31 December 2007 of singleton children to mothers in Scotland who had previously had a first child born by CS. Using available health records, they categorized these second births into scheduled repeat CS (assumed to be planned), unscheduled repeat CS, and VBAC. They then examined the childrens’ health records until 31 January 2015 and looked for correlations between type of birth and the following outcomes: obesity at age five years, hospitalization with asthma, prescription of a salbutamol inhaler (an asthma medication) at age five, hospitalization with inflammatory bowel disease, type 1 diabetes, learning disability, cerebral palsy, cancer, and death.

Of 40,145 births that took place, 44.6% were scheduled repeat CS, 22.1% were unscheduled repeat CS, and 33.3% were VBAC. The only consistent difference the researchers found between repeat CS (scheduled or unscheduled) and VBAC was a slightly elevated risk for hospitalization with asthma in children born by CS. They felt that this was not clinically significant, especially as there was no difference in the rate of salbutamol inhaler prescription. The risk of obesity at age five was similar between children born by repeat CS (scheduled or unscheduled) and those born by VBAC. Learning disability and death were more common following unscheduled repeat CS, but not scheduled repeat CS, than following VBAC.

**What Do These Findings Mean?:**

Overall, the right decision about a planned birth mode, vaginal or cesarean, depends on the individual case of mother and child. If mothers with a previous CS plan a vaginal birth, they should do so in a hospital that is prepared to perform a CS at short notice in case of scar rupture. The findings here suggest that there are no substantially worse outcomes associated with planned repeat cesarean births. They therefore support a process of planning birth after a previous CS that reflects a woman’s values and preferences. Whether the births analyzed in this study were initially planned to be vaginal or CS is not known. The researchers assumed an intended CS was one that was scheduled ahead of time and performed on the scheduled date. All other CSs were categorized as unplanned and likely represent a mix of emergency CS ahead of a planned CS and emergency CS after complications during a planned vaginal birth. The reasons for the observed higher risk of learning disability and death following unscheduled repeat CS are not clear because the records did not include the indication (i.e., the medical reason) for a CS, but the results are consistent with the known higher risk of an emergency CS to mothers and children.

The risks and benefits of different birth modes, especially in view of exposure to the mother’s microbiome, are active areas of research. The results here disagree with some earlier studies in different settings, but these were unable to adjust for some factors that can affect child health that are taken into account here, such as whether mothers were obese, smoked, or had asthma. The findings of this study are consistent with a study by the same researchers that analyzed all singleton first births from the Scottish health records and found slightly increased asthma rates, but no increased obesity or type 1 diabetes, in young children who had been delivered by CS compared with those who had a vaginal birth.

**Additional Information:**

This list of resources contains links that can be accessed when viewing the PDF on a device or via the online version of the article at http://dx.doi.org/10.1371/journal.pmed.1001973.

- The Royal College of Obstetricians and Gynaecologists in the UK has a practice guideline and patient information leaflet on birth after cesarean section
- The American Congress of Obstetricians and Gynecologists has a practice bulletin for vaginal birth after previous cesarean delivery, women’s health resources related to vaginal birth after cesarean, and women’s health resources related to cesarean section in general
- Wikipedia has a page on delivery after previous cesarean section (note that Wikipedia is an online encyclopedia that anyone can edit)
- The Royal Australian and New Zealand College of Obstetricians and Gynaecologists has a statement on birth after previous cesarean section
- The Institute of Obstetricians and Gynaecologists of the Royal College of Physicians of Ireland has a clinical practice guideline on delivery after cesarean section

## Introduction

Cesarean section (CS) accounts for a quarter of UK births, and between 1% and 52% of births in countries across the globe, with a previous CS being the leading indication [1,2]. Because there are few absolute indications for a repeat CS, most women are eligible to attempt vaginal birth after CS (VBAC), but uptake of attempted VBAC is variable, ranging from 9% in the US (2007 data) to 52% in the UK (2005–2012 data) [3–5]. In addition to the preference of the individual woman, multiple social, psychological, and medical considerations drive the decision either to opt for a repeat CS or to aim for VBAC [6,7]. Offspring consequences of alternative birth modes after CS may influence both the woman’s decision and clinician advice, but because of a lack of primary studies, data on childhood outcomes are limited and are mainly extrapolated from other populations [8].

Attempting VBAC after a previous CS carries a 0.5% risk of uterine scar rupture, with affected offspring having a 6% risk of hypoxic brain injury and a 1% risk of death [9]. Intrapartum hypoxic brain injury can lead to cerebral palsy, accounting for 2%–10% of all cerebral palsy cases [10,11]. While planned repeat CS avoids such risk, offspring miss out on potentially beneficial processes of labour, with or without vaginal delivery, including exposure to maternal bowel flora. Experimental data suggest that such exposure is required for normal development of the baby’s gut microbiome, and that CS, by preventing such exposure, adversely affects offspring immune function and epigenetic activity [12–15]. This may explain the increased relative risk (RR) of asthma-related illness (RR 1.17), obesity (RR 1.34), type 1 diabetes (RR 1.23), inflammatory bowel disease (RR 1.29), and cancer (RR 2.10) reported in CS-delivered offspring [16–20]. Hence, in order to ensure that birth choices are informed, research is needed into long-term health outcomes in children born by CS, with particular focus on those born to women with a history of CS [21]. We aimed to use a national birth cohort to compare the risk of adverse childhood health outcomes following planned CS and unscheduled CS with that following vaginal birth in women who had a previous CS, and to compare outcomes following planned repeat CS with those of unscheduled repeat CS to explore the role of exposure to labour.

## Methods

This study received approval from the North of Scotland Research Ethics Committee, the Privacy Advisory Committee of Information Services Division (ISD) Scotland, and the Caldicott Guardians for NHS Scotland Health Boards. Consent was not obtained from individual study participants as data were used and analysed anonymously.

This historical cohort study identified all term, singleton second deliveries occurring between 1 January 1993 and 31 December 2007 in Scotland, UK, to mothers with a history of a previous CS. Exclusion criteria included the following: stillbirths; offspring with missing data on year and month of delivery, mode of delivery, or sex; missing or implausible (>44 wk) data on gestation at delivery; maternal age less than 16 or over 53 y; and birthweight less than 1,500 g or over 5,500 g; exclusion criteria affected less than 0.1% of cases for each variable. Offspring were at least 6 y of age, if still alive, at the time of the study.

The study population was obtained from the Scottish Morbidity Record 02 (SMR02), which contains social, demographic, and clinical data on all deliveries in women discharged from maternity hospitals in Scotland for over three decades. Data are entered by dedicated data entry staff. SMR02 quality assurance assessment has demonstrated accurate matching of variables with case records in 98% of individuals for offspring sex, date of delivery, and birthweight; 90%–94% of individuals for maternal smoking status, estimated gestation, and pregnancy number; and 97% of individuals for CS as mode of delivery [22]. Mode of birth is recorded in SMR02 using the Office of Population Censuses and Surveys’ Classification of Surgical Operations, 4th revision.

Using SMR02 as the base population, seven further national databases were record-linked to SMR02 by Information Services Division (ISD) Scotland. (1) The Child Health Systems Programme School routinely records several features of child health measured in school including body mass index (BMI) centile adjusted for age, based upon height and weight measured in primary one (first year of compulsory fulltime education in the UK), when pupils’ average age is 5 y (range 4.5–6.25). Valid BMI recordings on 80% of first-born primary one pupils in Scotland were available for 1 January 2004–31 December 2007 [23,24]. This database provided data on childhood obesity (BMI over 95th centile). (2) The Scottish Morbidity Record 01 contains routinely recorded main condition diagnosed at discharge (using ICD-10 codes) from all acute admissions to hospital in Scotland, with validity checks suggesting 87% accuracy [25]. This database provided outcome data on asthma and inflammatory bowel disease diagnoses at discharge from hospital. (3) The Prescribing Information System is an electronic database of all filled community prescriptions issued by NHS Scotland since 1 January 1993, with a unique patient identifier (community health index number) recorded since 1 April 2009 [26]. This database provided confounder and outcome data on prescriptions for maternal and offspring salbutamol inhalers, respectively, from 1 April 2009–1 April 2013. (4) The Scottish Care Information Diabetes Collaboration is a clinical database that records details of all diabetes diagnoses registered with all Scottish health boards, with validity checks suggesting that 98% of type 1 diabetes cases are recorded accurately [27]. This database provided maternal and offspring diabetes diagnoses. (5) The Support Needs System is a clinical database used routinely by community paediatric service providers in Scottish health board areas, with both the Lothian and Grampian areas having utilised the system consistently since 2002. It contains details, including diagnoses, for all children who require additional support for their education or health, e.g., therapy or equipment, unless parental consent for recording of their data is withdrawn (affecting less than 1% in Grampian [personal communication, J. Crum, NHS Grampian, 10 November 2015]). This database provided details of children with a learning disability or cerebral palsy. (6) The Scottish Cancer Registry contains records of all cancer diagnoses in Scotland. It obtains data by screening datasets, administrative data systems, records of deaths, and community prescribing records, with 64%–100% completeness reported for various cancer types [28]. (7) The National Records of Scotland is a mandatory dataset compiled from all birth and death certificates in Scotland. This dataset provided details of the death of any offspring studied.

Mode of delivery (repeat CS or VBAC) was ascertained from SMR02. CSs were considered “planned” if the type of CS recorded in SMR02 was “scheduled”, while “unscheduled” procedures comprised all CSs not coded as “scheduled”. Scheduled CS is defined by ISD as a CS “performed during the day, with both staff and patient fully prepared”, reflecting a procedure that is planned in advance and expected (by the woman and health professionals) to occur on that day ([29]; personal communication, A. Duffy, ISD Scotland, 3 November 2015).

In the primary analysis, the risks of adverse childhood health outcomes following (1) planned repeat CS and (2) unscheduled repeat CS in term, second-born, singleton infants were compared to those in a group following a VBAC using a three-level categorical variable to represent type of birth in the model. In order to further explore the potential for exposure to labour to be either harmful or beneficial, an additional analysis compared outcomes following planned repeat CS with those following unscheduled repeat CS only. Acknowledging that, in reality, women may plan but not achieve VBAC, a third analysis was conducted comparing outcomes in offspring delivered by planned repeat CS with a combined group delivered by the alternative of unscheduled repeat CS or VBAC, as a proxy intended-birth-mode comparison.

Outcomes studied included obesity at age 5 y, asthma diagnosis at hospital discharge, salbutamol inhaler prescription at age 5 y, inflammatory bowel disease diagnosis at hospital discharge, type 1 diabetes, learning disability, cerebral palsy, cancer, and death. As death had the potential to be related to indications for CS or complications of delivery, which are concentrated in the first year of life, risk of death up to age 1 y was also assessed.

The full study cohort was utilised to analyse the outcomes hospitalisation with asthma, hospitalisation with inflammatory bowel disease, type 1 diabetes, cancer, and death, providing up to 21 y of offspring follow-up. For analysis of obesity at age 5 y, outcome data were available in 80% of cases from 2009 onwards, and therefore only births from 2004 to 2007 with complete outcome data were utilised. Collection of offspring BMI data varied by health board, with the number of health boards collecting data, and the proportion of completeness of such data within each health board, increasing incrementally over the study period. For analysis of salbutamol inhaler prescription, outcome data from 1 April 2009 to 1 December 2012 were utilised. To assess risk of salbutamol inhaler prescription at age 5 y, births occurring from 1 January 2004 to 31 December 2007 were included. For analysis of risk of learning disability and cerebral palsy, only offspring delivered in Lothian and Grampian were included, using a birth cohort from 1 January 1997 to 31 December 2007 that included school-aged children with relevant diagnoses consistently recorded on the Support Needs System from 1 January 2002 onwards.

The study sample allowed 99% power to detect a difference of 4% at a significance level of 5% in the incidence of the outcome obesity at age 5 y (population incidence 10%) following planned repeat CS delivery compared to VBAC [30].

For each of the comparison groups, continuous variables were summarised using mean and standard deviation or median and interquartile range, depending upon their distributions. The Student’s *t* test and Mann—Whitney *U* test were used to make comparisons between the groups. All hypothesis tests involved *p*-values that were two-sided at a significance level of 5%.

Hazard ratios (HRs) were calculated for the outcomes hospitalisation with asthma, hospitalisation with inflammatory bowel disease, type 1 diabetes, cancer, and death using Cox proportional hazards survival analysis. Where the number of events was at least 10-fold higher than the number of potential confounders to be included in the models, adjusted HRs were calculated. The proportional hazards assumption of the Cox proportional hazards model was tested using the plot of the log of the negative log of the survival function against log of time for each comparison group. The survival analysis starting point was the date of delivery, and the end point was either the end of follow-up or the event of interest. All patients whose end point was not the event of interest were censored.

Because outcome data were available for salbutamol inhaler prescription and offspring obesity for only a limited time period, adjusted odds ratios (ORs) were calculated for these outcomes at the age of 5 y using binary logistic regression. As date of diagnosis of learning disability and cerebral palsy was not available—and the majority of these diagnoses would be made prior to the offspring’s entry into the Support Needs System—binary logistic regression was also used to assess risk of learning disability and cerebral palsy. Model goodness of fit was assessed using the Hosmer—Lemeshow goodness of fit test.

Characteristics of cases with missing data were compared with those of cases with observed data. Multiple imputation was carried out, which assumes that the incomplete baseline data are missing at random (rather than missing completely at random) [31]. This means that the missingness of the baseline characteristics is assumed to occur at random, conditional upon the observed covariates. The results of the comparison of cases with and without missing data are provided in S1 Table. The pattern of missing data was explored and found to be non-monotone. Therefore a Markov chain Monte Carlo method was used to perform multiple imputation for the missing values relating to maternal Carstairs decile (deprivation status), maternal smoking status, breastfeeding at 6 wk, and maternal BMI. All available covariate and outcome variables were included in the imputation model (list provided in S2 Table), and continuous variables that were not normally distributed were log transformed to ensure that the assumption of normal distribution was met. Ten imputed datasets, considered enough to obtain reliable estimates for the effect of maternal BMI, were generated, and every statistical model was fitted to each. The estimates of effect and covariances from each imputed dataset were combined to produce inferential results.

Covariates adjusted for in each analysis are as follows: all models included maternal age, gestation at birth, maternal Carstairs decile, maternal smoking status, birthweight, year of delivery, male infant, and breastfeeding at 6 wk of age; models used to estimate offspring risk of asthma and salbutamol inhaler prescriptions also included maternal salbutamol inhaler prescription; risk estimates of offspring obesity at age 5 y also incorporated maternal BMI; and risk of type 1 diabetes was estimated with additional adjustment for maternal type 1 diabetes. All potential confounders adjusted for in the analyses were prespecified based upon expert knowledge and published literature. All statistical analyses were conducted using SPSS, version 22.

The analysis performed differed from that specified in the study protocol (S2 Text) in order to comply with reviewer recommendations as follows: a three-level variable was used to represent birth type in a model in which both planned repeat CS and unscheduled repeat CS were compared to VBAC as the referent, a direct comparison of outcomes following emergency CS and VBAC was performed, and the risk estimates for learning disability and cerebral palsy were calculated using binary logistic regression instead of survival analysis.

## Results

**Fig 1:**
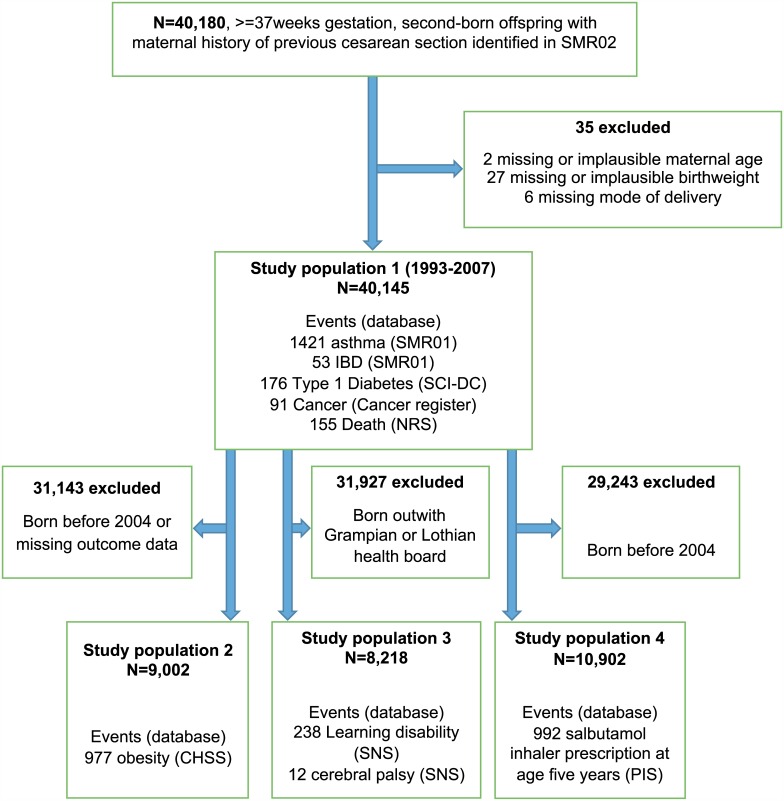
Cohort selection process, linked databases, total populations, and event counts. CHSP, Child Health Systems Programme; IBD, inflammatory bowel disease; NRS, National Records of Scotland; PIS, Prescribing Information System; SCI-DC, Scottish Care Information Diabetes Collaboration; SMR01, Scottish Morbidity Record 01; SNS, Support Needs System.

In total, 40,145 live-born singleton infants at or beyond 37 wk gestation delivered between 1 January 1993 and 31 December 2007 to women with a history of one previous CS were included in the analysis. The cohort derivation process is outlined in Fig 1. Data were missing in relation to maternal Carstairs decile (*n* = 79, 0.2%), maternal smoking status (*n* = 4,069, 10%), breastfeeding at 6 wk (*n* = 14,966, 37%), and maternal BMI (*n* = 34,527 in total cohort, *n* = 4,436 [49%] in cohort in which maternal BMI was utilised).

Of the 40,145 offspring, 17,919 (44.6%) were delivered by planned repeat CS, 8,847 (22.1%) were delivered by unscheduled repeat CS, and 13,379 (33.3%) were delivered by VBAC. The planned repeat CS rate increased across the study period from 36% to 57% among singleton second pregnancies at 37 wk or beyond. Median duration of follow-up of the full cohort was 165 mo (interquartile range 119–211).

Demographic and clinical characteristics of the planned and unscheduled repeat CS groups compared to the VBAC group are presented in Table 1.

**Table 1:** Demographic characteristics of the planned and unscheduled repeat cesarean groups compared to the VBAC group.

Characteristic	VBAC (*n* = 13,379)	Unscheduled Repeat CS (*n* = 8,847)	*p*-Value	Planned Repeat CS (*n* = 17,919)	*p*-Value
Maternal age, in years	29.57 (5.00	30.60 (4.92)	<0.01*	30.97 (5.00)	**<0.01** *
Maternal BMI, in kg/m^2^ ^†^	24.9 (22.3–28.2)	26.2 (23.3–30.5)	<0.01^‡^	27.1 (23.6–32.0)	**<0.01** ^‡^
Gestation, in weeks	39.71 (1.17)	39.57 (1.35)	<0.01*	38.75 (1.02)	**<0.01** *
Maternal Carstairs decile** ^§^	5 (3–8)	6 (3–8)	0.10^‡^	5 (3–8)	0.13^‡^
Maternal smoker^§^	2,892 (23.8%)	1,697 (21.3%)	<0.01^||^	2,850 (17.9%)	**<0.01** ^||^
Maternal salbutamol prescription	2,085 (15.6%)	1,587 (17.9%)	<0.01^||^	3,137 (17.5%)	**0.01** ^||^
Maternal type 1 diabetes	42 (0.3%)	67 (0.8%)	<0.01^||^	216 (1.2%)	**<0.01** ^||^
Birthweight, in grams	3,437 (469)	3,549 (549)	<0.01*	3,505 (507)	**<0.01** *
Year of delivery	1999 (1995–2003)	2000 (1996–2004)	<0.01^‡^	2001 (1997–2005)	**<0.01** ^‡^
Male offspring	6,732 (50.3%)	4,101 (46.4%)	<0.01^||^	9,047 (50.5%)	0.13^||^
Breastfeeding at 6 wk of age^§^	3,057 (39.0%)	2,020 (37.3%)	0.04^||^	3,835 (32.2%)	**<0.01** ^||^

Data are given as mean (standard deviation), median (interquartile range), or number (percent). Bold text indicates statistically significant findings at the 5% level.

*Student’s *t* test.

^†^Complete case data from 2004–2007 cohort.

^‡^Mann—Whitney *U* test.

**Calculated based upon adult male unemployment, lack of car ownership, low social class (based upon occupation), and overcrowding.

^§^Complete case data.

^||^Chi-squared test.

Women who delivered by unscheduled repeat CS were on average 1 y older and had a slightly higher BMI than those in the VBAC group. Deprivation level as measured by Carstairs decile was higher, but smoking rates were lower, in the unscheduled repeat CS group. Offspring delivered by unscheduled repeat CS were slightly heavier than the offspring delivered by VBAC. Comparison between the planned repeat CS and VBAC groups demonstrated that women in the planned repeat CS group were 1.5 y older, had a slightly higher BMI, were less likely to smoke, and were more likely to have type 1 diabetes. Offspring delivered by planned repeat CS were more likely to be delivered at an earlier gestational age, be heavier at birth, and be less likely to be breastfed at 6 wk of age compared to offspring delivered by VBAC.

Offspring childhood health outcomes following (1) planned repeat CS and (2) unscheduled repeat CS compared to VBAC are presented in Table 2.

**Table 2:** Offspring health outcomes after planned repeat cesarean and unscheduled repeat cesarean compared with after VBAC.

Outcome	VBAC Group (Reference Category)		Unscheduled Repeat CS Group				Planned Repeat CS Group			
	*n* Outcome Events/Total *N*	Percent	*n* Outcome Events/Total *N*	Percent	Unadjusted Risk of Outcome	Adjusted Risk of Outcome	*n* Outcome Events/Total *N*	Percent	Unadjusted Risk of Outcome	Adjusted Risk of Outcome
Obesity at age 5 y	169/2,254	7.5%	234/1,996	11.7%	**1.64 (1.33–2.02**)	1.10 (0.81–1.49)* ^†^	574/4,752	12.1%	**1.70 (1.42–2.03)**	1.18 (0.97–1.44)* ^†^
Salbutamol inhaler prescription at age 5 y	229/2,711	8.4%	222/2,375	9.3%	1.12 (0.92–1.36)	1.08 (0.88–1.31)* ^‡^	541/5,816	9.3%	1.11 (0.95–1.31)	1.04 (0.88–1.24)* ^‡^
Hospitalisation with asthma	442/13,379	3.3%	327/8,847	3.7%	**1.17 (1.02–1.35)**	**1.18 (1.05–1.33)** * ^‡^	652/17,919	3.6%	**1.18 (1.02–1.35)**	**1.24 (1.09–1.42)** * ^‡^
Hospitalisation with inflammatory bowel disease	21/13,379	0.2%	15/8,847	0.2%	1.30 (0.67–2.53)	—	17/17,919	0.1%	0.82 (0.43–1.56)	—
Type 1 diabetes mellitus	68/13,379	0.5%	33/8,847	0.4%	0.78 (0.51–1.18)	0.71 (0.47–1.08)* ^§^	75/17,919	0.4%	0.91 (0.66–1.27)	0.71 (0.49–1.04)* ^§^
Learning disability	66/2,859	2.3%	73/1,971	3.7%	**1.65 (1.19–2.31)**	**1.64 (1.17–2.29)** *	99/3,388	2.9%	1.35 (0.99–1.84)	1.17 (0.82–1.65)*
Cerebral palsy	3/2,859	0.1%	5/1,971	0.3%	2.44 (0.58–10.22)	—	4/3,388	0.1%	1.17 (0.26–5.23)	—
Cancer	40/13,379	0.3%	18/8,847	0.2%	0.72 (0.41–1.25)	—	33/17,919	0.2%	0.67 (0.42–1.06)	—
Death	53/13,379	0.4%	44/8,847	0.5%	1.30 (0.87–1.95)	**1.50 (1.00–2.25)** *	58/17,919	0.3%	0.87 (0.60–1.27)	1.01 (0.66–1.53)*
Death up to 1 y of age	29/13,397	0.2%	23/8,837	0.3%	1.20 (0.69–2.07)	1.36 (0.78–2.37)*	26/17,919	0.1%	0.67 (0.39–1.14)	0.77 (0.44–1.36)*

Data are from a model with type of birth as a three-level categorical variable, with VBAC as the reference category. Outcome risks are HR (95% CI), except for obesity at age 5 y, salbutamol inhaler prescription at age 5 y, learning disability, and cerebral palsy, for which the outcome risks are OR (95% CI). Blank cells indicate adjusted analyses not performed due to small number of events. Bold text indicates statistically significant findings at the 5% level.

*Adjusted for maternal age, gestation at birth, maternal Carstairs decile, maternal smoking status, birthweight, year of delivery, male infant, and breastfeeding at 6 wk.

^†^Adjusted for maternal BMI.

^‡^Adjusted for maternal salbutamol prescription.

^§^Adjusted for maternal type 1 diabetes.

While crude analysis suggested a 64% increased risk of obesity at age 5 y following planned repeat CS compared with VBAC, there were no statistically significant differences demonstrated following either planned or unscheduled repeat CS compared with VBAC, once the analysis was adjusted for potential confounders. Risk of hospitalisation with asthma was significantly higher following both unscheduled repeat CS (18% risk increase) and planned repeat CS (24% risk increase) compared with VBAC. Risk of learning disability was 64% higher in the unscheduled repeat CS group compared with the VBAC group, but no significant difference was identified when comparing planned repeat CS with VBAC. Death in childhood was 50% more likely in offspring delivered by unscheduled repeat CS compared with those delivered by VBAC (absolute difference in risk 0.1%), although no difference was apparent in the analysis of death risk up to 1 y of age. There were no significant differences in risk of salbutamol inhaler prescription at age 5 y, inflammatory bowel disease, type 1 diabetes, cerebral palsy, or cancer when comparing either CS group with VBAC.

Due to the risk of model overfitting in the presence of few events, no adjustments were made for potential confounders in the analyses relating to the inflammatory bowel disease, cerebral palsy, and cancer outcomes [32].

Outcomes of planned repeat CS compared with unscheduled repeat CS are presented in Table 3. Crude analyses demonstrated a significantly reduced risk of death of offspring delivered by planned repeat CS, with absolute difference of 0.2%. However, in adjusted analyses, there were no statistically significant differences in risk for any of the outcomes measured.

**Table 3:** Offspring health outcomes comparing planned repeat cesarean with unscheduled repeat cesarean delivery.

Outcome	Planned Repeat CS Group		Unscheduled Repeat CS Group		Unadjusted Risk of Outcome	Adjusted Risk of Outcome
	*n* Outcome Events/Total *N*	Percent	*n* Outcome Events/Total *N*	Percent		
Obesity at age 5 y	574/4,752	12.1%	234/1,996	11.7%	1.04 (0.88–1.22)	0.97 (0.76–1.25)* ^†^
Salbutamol inhaler prescription at age 5 y	541/5,816	9.3%	222/2,375	9.3%	1.00 (0.95–1.05)	0.96 (0.81–1.14)* ^‡^
Hospitalisation with asthma	652/17,919	3.6%	327/8,847	3.7%	1.01 (0.88–1.15)	1.02 (0.89–1.18)* ^‡^
Hospitalisation with inflammatory bowel disease	17/17,919	0.1%	15/8,847	0.2%	1.00 (0.56–1.77)	—
Type 1 diabetes mellitus	75/17,919	0.4%	33/8,847	0.4%	1.17 (0.78–1.77)	1.15 (0.74–1.78)* ^§^
Learning disability	99/3,388	2.9%	73/1,971	3.7%	0.81 (0.60–1.10)	0.76 (0.55–1.05)*
Cerebral palsy	4/3,388	0.1%	5/1,971	0.3%	0.46 (0.13–1.73)	—
Cancer	33/17,919	0.2%	18/8,847	0.2%	0.92 (0.52–1.64)	—
Death	58/17,919	0.3%	44/8,847	0.5%	**0.66 (0.45–0.98)**	0.67 (0.44–1.01)*

Outcome risks are HR (95% CI), except for obesity at age 5 y and salbutamol inhaler prescription at age 5 y, for which the outcome risks are OR (95% CI). Blank cells indicate adjusted analyses not performed due to small number of events. Bold text indicates statistically significant findings at the 5% level.

*Adjusted for maternal age, gestation at birth, maternal Carstairs decile, maternal smoking status, birthweight, year of delivery, male infant, and breastfeeding at 6 wk.

^†^Adjusted for maternal BMI.

^‡^Adjusted for maternal salbutamol prescription.

^§^Adjusted for maternal type 1 diabetes.

Covariates adjusted for in the analyses are indicated in the footnote of Table 3. No adjustments were made in the analyses relating to inflammatory bowel disease, cerebral palsy, or cancer.

Results of the comparison between planned CS and all unscheduled births are included in S3 Table. This comparison demonstrates that there are no statistically significant differences in the studied offspring outcomes between the two groups.

The multivariable analyses obtained using imputed data and complete cases only demonstrated that, whilst the effect sizes were similar, risk of learning disability became significantly decreased following planned repeat CS compared with all unscheduled births in the complete case analysis, as shown in S4 Table.

## Discussion

In this analysis of over 40,000 offspring in a Scottish cohort, we have demonstrated, to our knowledge for the first time, a positive association between repeat CS delivery, whether planned or unscheduled, and offspring hospitalisation with asthma, but we found no difference in risk of offspring obesity in the CS-delivered offspring. Learning disability and death were also more common in offspring born by unscheduled repeat CS compared with VBAC. The absolute difference of only 0.3% in prevalence of hospitalisation with asthma between offspring delivered by planned repeat CS and VBAC has minimal clinical significance and, if causal, would require 298 successful VBACs to evade one case of hospitalisation with asthma. The increased risk of learning disability and death following unscheduled repeat CS may reflect the risk associated with labour in the context of a previous cesarean scar, but risk of confounding by indication cannot be ruled out.

The magnitude of the association of planned repeat CS or unscheduled repeat CS with offspring hospitalisation with asthma compared to that of VBAC is consistent with published reports on asthma risk following any (planned or unplanned) CS in unselected populations [33]. With the link observed after both types of CS, and no such link demonstrated when comparing planned with unscheduled repeat CS, the increased asthma risk could be explained by a lack of protective effect of the exposure to maternal bowel flora during vaginal passage, rather than such exposure following membrane rupture alone, as would often precede unscheduled CS. Such an explanation is speculative at this stage, as no data on actual exposure to maternal bowel flora were available within the context of this study. An absence of association between planned repeat CS and prescription of the first-line treatment for asthma—salbutamol inhaler—suggests that any causal link between planned repeat CS and asthma may be specific to more severe asthmatic phenotypes. An alternative explanation for these arguably conflicting results involves potential confounding by maternal health-related behaviour, as influences that shape a choice to have a planned repeat CS may be in common with those that encourage a parent to take offspring to hospital for treatment of asthma.

Studies of offspring obesity following planned CS specifically, have, in keeping with our findings, not identified any significant association in adjusted analyses [34–38]. Such studies include our related work on childhood health outcomes in first-born offspring in a Scottish cohort [38]. These reports conflict with published literature showing an increased risk of obesity following any CS, but this difference is likely explained by the nature of data sources (including our own) that allow differentiation between planned and unplanned CS. Such datasets tend to have more extensive covariate data such that crude associations can be shown to disappear in adjusted analyses [34–36,39].

Our findings of no significant associations between planned repeat CS and the immune-mediated conditions type 1 diabetes, inflammatory bowel disease, and cancer are consistent with contemporary cohort studies of offspring health following planned CS comprising in excess of 2 million offspring each [19,40,41]. While these findings contrast with earlier studies comparing offspring health following any CS with that following vaginal birth, the discrepancies are likely explained by the availability of detailed contemporary data on both the nature of CS and potential confounders. Further explanations in the context of type 1 diabetes and cancer include the reduced risk of selection bias in recent studies due to the use of sibling analysis and cohort (rather than case—control) study designs, respectively [18,20,42].

With regard to risk of learning disability or cerebral palsy in offspring delivered by planned repeat CS, there are no identified published data on this relationship despite recognition that long-term outcomes may be affected, particularly by hypoxic brain injury caused by scar rupture in labour [8]. Our findings of no significant differences in risks between planned repeat CS and unscheduled birth after CS (both unscheduled repeat CS and VBAC) appear somewhat reassuring. However, the increased risk of learning disability in offspring delivered by unscheduled repeat CS compared with VBAC highlights a substantial disparity in outcomes depending on whether an apparent plan for VBAC is successful. The lack of a significant difference in risk of cerebral palsy in all comparisons must be interpreted with caution, given the low number of events.

This study found that the risk of death of offspring following planned repeat CS did not differ significantly from that following unscheduled repeat CS, but with confidence intervals that only just crossed unity, there is justified speculation that a larger cohort would have yielded significant results in favour of planned repeat CS. This view is supported by the results of previous larger studies from Scotland and the US in which planned repeat CS appeared protective against perinatal death [9,43]. Our finding of an increased risk of death following unscheduled repeat CS compared with VBAC serves to highlight this issue further, as the majority of unscheduled repeat CSs are expected to have involved failed VBAC attempts. The risk of death beyond the neonatal period has not to our knowledge been previously reported in relation to birth after CS, and this study provides reassurance that survival to 21 y of age did not differ significantly whether birth after CS was by planned CS or otherwise.

Our study demonstrates a novel use of national data to assess childhood outcomes relating to birth mode after CS, ensuring results that are applicable to term pregnancies after one CS. Acknowledging that in aiming for VBAC rather a planned repeat CS, vaginal birth is not guaranteed, this study included an analysis that grouped both VBAC and unscheduled repeat CS births together as “unscheduled births” to more closely resemble real-life scenarios. Recognising that potential risk associated with CS births, such as childhood asthma, may differ according to degree of exposure to labour processes and maternal bowel flora, this study included direct comparisons of outcomes between planned repeat CS and each of unscheduled repeat CS and VBAC, enabling any dose—response effect to be identified. The comparison between planned CS and unscheduled CS, and the additional analysis comparing planned CS with all other births, is original, as previous studies of offspring health after CS have either grouped all CSs together and compared outcomes with those of vaginal birth or compared outcomes of each type of CS (planned and unscheduled) with those of vaginal birth only [16,19,33,34,37,40,44]. The population studied ensured clinical relevance, as most women who have the opportunity to pursue their favoured birth mode after CS will have singleton pregnancies that reach term. The study design minimised the potential for confounded associations between mode of birth and offspring health outcomes by adjusting for multiple maternal, demographic, and offspring characteristics. To deal specifically with the potential effect of time trends in both CS practice and background morbidity rates, year of delivery was included in all adjusted models. Missing data were imputed using multiple imputation, which allowed us to include individuals in the analysis who had incomplete data. This increases the statistical power to identify any significant associations. A complete case analysis would have considerably reduced the size of the sample and the resulting precision of the analyses. The sample size utilised as a result of imputing missing data allowed adequate power to assess common outcomes including obesity and salbutamol inhaler prescription, despite offspring not resident in Scotland being missed from follow-up. Furthermore, since differences in characteristics were shown to exist between patients with complete and incomplete data, multiple imputation addresses any potential biases that may have occurred due to excluding patients with data missing at random. The findings of this study are considered to be generalisable to countries with similar CS rates and background prevalence rates of the outcomes studied.

An important limitation of the study involved the lack of data on indication for CS or cause of death, such that the potential for residual confounding remains. This is particularly relevant where a signal of fetal compromise prompted a planned or unscheduled repeat CS, following which the infant may have died from a related cause. It is also recognised that by analysing offspring according to their actual mode of birth, the outcomes following intended birth mode may not be represented. It is important to note that women and health professionals have greatest control over the intended, rather than the actual, mode of birth, such that the findings cannot be directly applied to mode of delivery decisions. In addition, missing outcome data on offspring learning disability and cerebral palsy (from all except the two health boards used for the analysis of these outcomes) limited the power to detect clinically significant effects, while the fact that 20% of offspring from across Scotland were missing obesity outcome data had the potential to bias the study findings.

Our study demonstrated a weak, clinically insignificant association between planned repeat CS birth and offspring hospitalisation with asthma, consistent with existing literature. There was a lack of association, positive or negative, between planned repeat CS and other clinically important adverse health outcomes in childhood. An increased risk of learning disability and death following unscheduled repeat CS compared with VBAC could reflect the known risk of scar rupture during a VBAC attempt, ultimately leading to an unscheduled CS and potential hypoxic brain injury or infant death [9]. However, unscheduled repeat CS being associated with the least favourable outcomes does not prove causality, as this mode of delivery may have been prompted by an underlying clinical problem. The disparity in outcomes following unscheduled repeat CS and successful VBAC support existing research, but require further investigation to relate outcomes to planned mode of birth. The lack of data on intended (rather than actual) mode of birth limits the direct application of these study findings to clinical practice, but women may be somewhat reassured by the apparent lack of risk to long-term offspring health following planned repeat CS specifically. This study may therefore support the process of planning birth after CS in a way that reflects women’s values and preferences.

## Supporting Information

S1 Table Comparison of case characteristics between cases with complete and those with missing data. (DOCX)

S2 Table Predictor variables included in the multiple imputation process. (DOCX)

S3 Table Comparison of offspring outcomes between planned repeat CS and unscheduled births (unscheduled repeat CS and VBAC). (DOCX)

S4 Table Complete case analysis of offspring health outcomes by mode of birth. (DOCX)

S1 Text Completed STROBE checklist. (DOC)

S2 Text Study protocol. (DOCX)

## Abbreviations

BMI: body mass index
CS: cesarean section
HR: hazard ratio
ISD: Information Services Division
OR: odds ratio
RR: relative risk
SMR02: Scottish Morbidity Record 02
VBAC: vaginal birth after cesarean section
