# Supplementary material for: Planned Repeat Cesarean Section at Term and Adverse Childhood Health Outcomes: A Record-Linkage Study
Source: PLoS Med. 2016 Mar 15;13(3):e1001973. doi: 10.1371/journal.pmed.1001973 (PMC4792387; doi:10.1371/journal.pmed.1001973)
Supplement: S1 Table — (DOCX) [file pmed.1001973.s001.docx]

S1 Table. Comparison of characteristics between cases with complete and those with missing data

S1A Table. Comparison of characteristics between cases with complete and those with missing data on maternal social class (Carstairs Decile)

| **Variable** | **Carstairs data present n (%)** | **Carstairs data missing n (%)** | **p-value** |
| --- | --- | --- | --- |
|  | **N=40066** | **N=79** |  |
| Planned repeat caesarean section delivery | 17882 (44.6) | 37 (46.8) | 0.78* |
| Gestation at delivery, mean (SD) | 39.3 (1.2) | 39.2 (1.3) | 0.61** |
| Maternal age, mean (SD) | 30.4 (5.0) | 31.0 (4.6) | 0.36** |
| Maternal smoker | 7427 (20.6) | 12 (16) | 0.40* |
| Maternal BMI, median (IQR) | 26.2 (23.1-30.5) | 24.3 (22.6-27.6) | 0.19~ |
| Male infant | 19665 (49.1) | 40 (50.6) | 0.87* |
| Birth weight of baby, mean (SD) | 3492.2 (506.5) | 3510.1 (542.4) | 0.75** |
| Infant feeding at 6-8 weeks of age | 8898 (35.4) | 14 (35.9) | 1* |
| Salbutamol inhaler use - child | 6615 (16.5) | 12 (15.2) | 0.87* |
| Salbutamol inhaler use - mother | 6803 (17) | 6 (7.6) | 0.04* |
| Hospitalisation with asthma | 1416 (3.5) | 5 (6.3) | 0.21† |
| Insulin prescription - child | 171 (0.4) | 0 | 1† |
| Insulin prescription - mother | 472 (1.2) | 0 | 1† |
| Inflammatory bowel disease as discharge diagnosis | 53 (0.1) | 0 | 1† |
| Childhood obesity | 2051 (11) | 10 (26.3) | 0.01* |
| Child on support needs system | 1045 (5.3) | 1 (2.6) | 0.72† |
| Cerebral palsy | 40 (0.1) | 0 | 1† |
| Cancer | 91 (0.2) | 0 | 1† |
| Time at risk of cancer, median (IQR) | 165 (119-211) | 154 (127-217) | 0.87~ |
| Time at risk of asthma, median (IQR) | 162 (114-208) | 150 (121-213) | 0.75~ |
| Time at risk of death, median (IQR) | 165 (199-211) | 154 (127-217) | 0.85~ |
| Time at risk of IBD, median (IQR) | 165 (119-211) | 154 (127-217) | 0.86~ |
| Year of delivery (IQR) | 2000 (1996-2004) | 2001 (1996-2003) | 0.77~ |
| Death | 155 (0.4) | 0 | 1† |

*calculated using Chi-squared test **calculated using independent samples t-test; ~calculated using Mann-Whitney test †calculated using Fisher’s exact test, SD=standard deviation, IQR=interquartile range, BMI=body mass index

S1B Table

Comparison of characteristics between cases with complete and those with missing data on maternal smoking status

| **Variable** | **Maternal smoking data present n (%)** | **Maternal smoking data missing n (%)** | **p-value** |
| --- | --- | --- | --- |
|  | **N=36076** | **N=4069** |  |
| Planned repeat caesarean section delivery | 15949 (44.2) | 1970 (48.4) | <0.01* |
| Gestation at delivery, mean (SD) | 39.26 (1.23) | 39.19 (1.24) | <0.01** |
| Maternal age, mean (SD) | 30.49 (5.01) | 29.77 (5.12) | <0.01** |
| Maternal deprivation category, median (IQR) | 6 (3-8) | 6 (3-8) | <0.01~ |
| Maternal BMI, median (IQR) | 26.2 (23.2-30.5) | 26.22 (22.5-32.1) | 0.93~ |
| Male infant | 17717(49.1) | 1988 (48.9) | 0.77* |
| Birth weight of baby, mean (SD) | 3494 (505.95) | 3476 (511.5) | 0.04** |
| Breast-feeding at 6-8 weeks of age | 8297 (36.1) | 615 (28.3) | <0.01* |
| Salbutamol inhaler use - child | 5920 (16.4) | 707 (17.4) | 0.12* |
| Salbutamol inhaler use - mother | 6087 (16.9) | 722 (17.7) | 0.16* |
| Hospitalisation with asthma | 1252 (3.5) | 169 (4.2) | 0.03* |
| Insulin prescription - child | 152 (0.4) | 19 (0.5) | 0.67* |
| Insulin prescription - mother | 421 (1.2) | 51 (1.3) | 0.63* |
| Inflammatory bowel disease as discharge diagnosis | 42 (0.1) | 11 (0.3) | 0.01* |
| Childhood obesity | 1904 (11.1) | 157 (10.1) | 0.25* |
| Child on support needs system | 928 (5.1) | 118 (7.1) | <0.01* |
| Cerebral palsy | 37 (0.1) | 3 (0.1) | 0.77* |
| Cancer | 78 (0.2) | 13 (0.3) | 0.26* |
| Time at risk of cancer, median (IQR) | 163 (119-207) | 203 (115-229) | <0.01~ |
| Time at risk of asthma, median (IQR) | 160 115-204) | 197 (109-227) | <0.01~ |
| Time at risk of death, median (IQR) | 163 (120-207) | 204 (115-229) | <0.01~ |
| Time at risk of IBD, median (IQR) | 163 (120-206) | 203 (115-203) | <0.01~ |
| Year of delivery, median (IQR) | 2000 (1997-2004) | 1997 (1995-2004) | <0.01~ |
| Death | 142 (0.4) | 13 (0.3) | 0.56* |

*calculated using Chi-squared test **calculated using independent samples t-test; ~calculated using Mann-Whitney test †calculated using Fisher’s exact test, SD=standard deviation, IQR=interquartile range, BMI=body mass index

S1C

Comparison of characteristics between cases with complete and those with missing data on breastfeeding status at six weeks of age

| **Variable** | **Maternal breastfeeding data present n (%)** | **Maternal breastfeeding data missing n (%)** | **p-value** |
| --- | --- | --- | --- |
|  | **N=25179** | **N=14966** |  |
| Planned repeat caesarean section delivery | 11928 (47.4) | 5991 (40) | <0.01* |
| Gestation at delivery, mean (SD) | 39.2 (1.2) | 39.3 (1.3) | <0.01** |
| Maternal age, mean (SD) | 30.7 (5.0) | 29.9 (5.0) | <0.01** |
| Maternal deprivation category, median (IQR) | 6 (3-8) | 5 (2-7) | <0.01~ |
| Maternal smoker | 4553 (19.8) | 2886 (22.1) | <0.01* |
| Male infant | 12378 (49.2) | 7327 (49.0) | 0.70* |
| Birth weight of baby, mean (SD) | 3496.0 (507.2) | 3486 (505.4) | <0.01** |
| Maternal BMI | 26.2 (23.2-30.8) | 25.9 (22.9-30.1) | 0.15 |
| Salbutamol inhaler use - child | 4253 (16.9) | 2374 (15.9) | <0.01* |
| Salbutamol inhaler use - mother | 4244 (16.9) | 2565 (17.1) | 0.47* |
| Hospitalisation with asthma | 759 (3.0) | 662 (4.4) | <0.01* |
| Insulin prescription - child | 95 (0.4) | 76 (0.5) | 0.06* |
| Insulin prescription - mother | 288 (1.1) | 184 (1.2) | 0.47* |
| Inflammatory bowel disease as discharge diagnosis | 27 (0.1) | 26 (0.2) | 0.10* |
| Childhood obesity | 1649 (10.9) | 412 (11.6) | 0.26* |
| Child on support needs system | 587 (3.7) | 459 (11.5) | <0.01* |
| Cerebral palsy | 20 (0.1) | 20 (0.1) | 0.13* |
| Cancer | 62 (0.2) | 29 (0.2) | 0.34* |
| Time at risk of cancer, median (IQR) | 149 (111-188) | 208 (145-237) | <0.01~ |
| Time at risk of asthma, median (IQR) | 146 (107-187) | 203 (137-236) | <0.01~ |
| Time at risk of death, median (IQR) | 149 (111-188) | 208 (145-237) | <0.01~ |
| Time at risk of IBD, median (IQR) | 149 (111-188) | 208 (145-237) | <0.01~ |
| Year of delivery, median (IQR) | 2001 (1998-2005) | 1996 (1994-2002) | <0.01~ |
| Death | 56 (0.2) | 99 (0.7) | <0.01~ |

*calculated using Chi-squared test **calculated using independent samples t-test; ~calculated using Mann-Whitney test †calculated using Fisher’s exact test, SD=standard deviation, IQR=interquartile range, BMI=body mass index

S1D Table

Comparison of case characteristics between cases with complete and those with missing data on maternal body mass index

| **Variable** | **Maternal BMI data present n (%)** | **Maternal BMI data missing n (%)** | **p-value** |
| --- | --- | --- | --- |
|  | **N=4566** | **N=4436** |  |
| Planned repeat caesarean section | 2454 (53.7) | 2298 (51.8) | 0.07* |
| Gestation at delivery, mean (SD) | 39.19 (1.16) | 39.23 (1.15) | 0.09** |
| Maternal age, mean (SD) | 31.01 (5.23) | 31.29 (5.23) | <0.01** |
| Maternal deprivation category, median (IQR) | 6 (3-8) | 5 (2-7) | <0.01~ |
| Maternal smoker | 737 (17.2) | 600 (15.9) | 0.10* |
| Male infant | 2267 (49.6) | 2189 (49.3) | 0.79* |
| Birth weight of baby, mean (SD) | 3523 (501.7) | 3533 (501.1) | 0.31** |
| Any breast feeding at 6-8 weeks of age | 1291 (32.7) | 1173 (39.9) | <0.01* |
| Salbutamol inhaler use - child | 895 (19.6) | 814 (18.3) | 0.13* |
| Salbutamol inhaler use – mother | 787 (17.2) | 7655 (17.2) | 0.99* |
| Hospitalisation with asthma | 82 (1.8) | 85 (1.9) | 0.96* |
| Insulin prescription - child | 14 (0.3) | 9 (0.2) | 0.33* |
| Insulin prescription - mother | 52 (1.1) | 55 (1.2) | 0.66* |
| Inflammatory bowel disease as discharge diagnosis | 1 (2.19e-4) | 0 (0) | 0.32* |
| Childhood obesity | 503 (11) | 474 (10.7) | 0.61* |
| Child on support needs system | 52 (1.1) | 92 (2.1) | <0.01* |
| Cerebral palsy | 1 (2.19e-4) | 1 (2.25e-4) | 0.98* |
| Cancer | 7 (0.2) | 7 (0.2) | 0.96* |
| Year of delivery | 2006 (2005-2007) | 2006 (2005-2007) | 0.04~ |
| Death | 0 | 0 | 1 |

*calculated using Chi-squared test **calculated using independent samples t-test; ~calculated using Mann-Whitney test †calculated using Fisher’s exact test, SD=standard deviation, IQR=interquartile range, BMI=body mass index
