# Supplementary material for: Planned Repeat Cesarean Section at Term and Adverse Childhood Health Outcomes: A Record-Linkage Study
Source: PLoS Med. 2016 Mar 15;13(3):e1001973. doi: 10.1371/journal.pmed.1001973 (PMC4792387; doi:10.1371/journal.pmed.1001973)
Supplement: S2 Table — (DOCX) [file pmed.1001973.s002.docx]

Supplementary Table File 2. Predictor variables included in the multiple imputation process

S2A Table. Predictor variables included in the multiple imputation process to impute missing values on maternal Carstairs decile:

| Variable Type | Predictor |
| --- | --- |
| Continuous | Gestation at delivery (weeks) |
|  | Maternal age at delivery (years) |
|  | Birth weight of baby (grams) |
|  | Maternal BMI (units) |
|  | Year of delivery (years) |
|  | Time at risk of cancer (months) |
|  | Time at risk of asthma (months) |
|  | Time at risk of death (months) |
|  | Time at risk of IBD (months) |
| Binary | Planned repeat caesarean section delivery |
|  | Maternal smoking |
|  | Male infant |
|  | Salbutamol inhaler use - child |
|  | Salbutamol inhaler use - mother |
|  | Asthma as hospital discharge diagnosis |
|  | Insulin prescription - child |
|  | Insulin prescription - mother |
|  | Inflammatory bowel disease as discharge diagnosis |
|  | Childhood obesity |
|  | Child on support needs system |
|  | Cerebral palsy |
|  | Cancer |
|  | Death |
|  | Breastfeeding |

S2B Table Predictor variables included in the multiple imputation process to impute missing values on maternal smoking status:

| Variable Type | Predictor |
| --- | --- |
| Continuous | Maternal Carstairs decile |
|  | Gestation at delivery (weeks) |
|  | Maternal age at delivery (years) |
|  | Birth weight of baby (grams) |
|  | Maternal BMI (units) |
|  | Year of delivery (years) |
|  | Time at risk of cancer (months) |
|  | Time at risk of asthma (months) |
|  | Time at risk of death (months) |
|  | Time at risk of IBD (months) |
| Binary | Planned repeat caesarean section delivery |
|  | Male infant |
|  | Salbutamol inhaler use - child |
|  | Salbutamol inhaler use - mother |
|  | Asthma as hospital discharge diagnosis |
|  | Insulin prescription - child |
|  | Insulin prescription - mother |
|  | Inflammatory bowel disease as discharge diagnosis |
|  | Childhood obesity |
|  | Child on support needs system¥ |
|  | Cerebral palsy¥ |
|  | Cancer |
|  | Death |
|  | Breastfeeding |

S2C Table. Predictor variables included in the multiple imputation process to impute missing values on breastfeeding status at six weeks of age:

| Variable Type | Predictor |
| --- | --- |
| Continuous | Maternal Carstairs decile |
|  | Gestation at delivery (weeks) |
|  | Maternal age at delivery (years) |
|  | Birth weight of baby (grams) |
|  | Maternal BMI (units) |
|  | Year of delivery (years) |
|  | Time at risk of cancer (months) |
|  | Time at risk of asthma (months) |
|  | Time at risk of death (months) |
|  | Time at risk of IBD (months) |
| Binary | Planned repeat caesarean section delivery |
|  | Maternal smoking |
|  | Male infant |
|  | Salbutamol inhaler use - child |
|  | Salbutamol inhaler use - mother |
|  | Asthma as hospital discharge diagnosis |
|  | Insulin prescription - child |
|  | Insulin prescription - mother |
|  | Inflammatory bowel disease as discharge diagnosis |
|  | Childhood obesity |
|  | Child on support needs system¥ |
|  | Cerebral palsy¥ |
|  | Cancer |
|  | Death |

S2D Table. Predictor variables included in the multiple imputation process to impute missing values on maternal body mass index

| Variable Type | Predictor |
| --- | --- |
| Continuous | Maternal Carstairs decile |
|  | Gestation at delivery (weeks) |
|  | Maternal age at delivery (years) |
|  | Birth weight of baby (grams) |
|  | Maternal BMI (units) |
|  | Year of delivery (years) |
|  | Time at risk of cancer (months) |
|  | Time at risk of asthma (months) |
|  | Time at risk of death (months) |
|  | Time at risk of IBD (months) |
| Binary | Planned repeat caesarean section delivery |
|  | Maternal smoking |
|  | Male infant |
|  | Salbutamol inhaler use - child |
|  | Salbutamol inhaler use - mother |
|  | Asthma as hospital discharge diagnosis |
|  | Insulin prescription - child |
|  | Insulin prescription - mother |
|  | Inflammatory bowel disease as discharge diagnosis |
|  | Childhood obesity |
|  | Child on support needs system¥ |
|  | Cerebral palsy¥ |
|  | Cancer |
|  | Death |
|  | Breastfeeding |
