# Supplementary material for: Planned Repeat Cesarean Section at Term and Adverse Childhood Health Outcomes: A Record-Linkage Study
Source: PLoS Med. 2016 Mar 15;13(3):e1001973. doi: 10.1371/journal.pmed.1001973 (PMC4792387; doi:10.1371/journal.pmed.1001973)
Supplement: S3 Table — (DOCX) [file pmed.1001973.s003.docx]

Supplementary Table File 3. Comparison of offspring outcomes between planned repeat CS and unscheduled births (unscheduled CS and VBAC)

| **Outcome** | **Unscheduled birth (unscheduled repeat CS and VBAC) offspring** |  | **Planned repeat CS delivered offspring** | | **Unadjusted risk of outcome** | **Adjusted risk of outcome** |
| --- | --- | --- | --- | --- | --- | --- |
|  | N outcome events/total N | % | n outcome events/total N | % |  |  |
| Obesity aged five years | 403/4250 | 9.5 | 574/4752 | 12.1 | OR **1.31 (1.15-1.50)** | OR 1.02 (0.83-1.26) †‡ |
| Salbutamol inhaler use aged 5 years | 451/5086 | 8.9 | 541/5816 | 9.3 | OR 1.11 (0.98-1.25) | OR 0.99 (0.87-1.14)†* |
| Hospitalisation with asthma <=30months of age* | 190/22226 | 0.9 | 167/17919 | 0.9 | HR 1.10 (0.88-1.34) | HR 1.23 (0.97-1.54)†* |
| Hospitalisation with asthma >30months of age* | 571/21963 | 2.6 | 476/17708 | 2.7 | HR 1.11 (0.98-1.25) | HR 1.11 (0.98-1.25) †* |
| Inflammatory bowel disease | 36/22226 | 0.2 | 17/17919 | 0.2 | HR 0.74 (0.41-1.31) |  |
| Type 1 diabetes mellitus | 101/22226 | 0.5 | 75/17919 | 0.4 | HR 1.00 (0.74-1.35) | HR 0.89 (0.64-1.25) †§ |
| Learning disability | 139/4830 | 2.9 | 99/3388 | 2.9 | OR 1.02 (0.78-1.32) | OR 0.94 (0.70-1.25) † |
| Cerebral palsy | 8/4830 | 0.2 | 4/3388 | 0.1 | OR 0.71 (0.21-2.37) |  |
| Cancer | 58/22226 | 0.3 | 33/17919 | 0.2 | HR 0.75 (0.49-1.15) | HR 0.66 (0.41-1.04) † |
| Death | 97/22226 | 0.4 | 58/17919 | 0.3 | HR 0.77 (0.56-1.07) | HR 0.84 (0.59-1.19) † |

*Due to suboptimal model fit when assessing risk of hospitalisation with asthma over the entire age range (up to 21 years), the analysis was split into events up to 30 months of age, and events beyond 30 months of age. †adjusted for maternal age, gestation at birth, maternal Carstairs decile, maternal smoking status, birthweight, year of delivery, male infant, breastfeeding at six weeks, ‡adjusted for maternal BMI. *adjusted for maternal salbutamol prescription. Bold text indicates statistically significant findings at the 5% level. Blank cells indicate adjusted analyses not performed due to small number of events.
