# Supplementary material for: Planned Repeat Cesarean Section at Term and Adverse Childhood Health Outcomes: A Record-Linkage Study
Source: PLoS Med. 2016 Mar 15;13(3):e1001973. doi: 10.1371/journal.pmed.1001973 (PMC4792387; doi:10.1371/journal.pmed.1001973)
Supplement: S4 Table — (DOCX) [file pmed.1001973.s004.docx]

Supplementary Table File 4. Complete case analyses of offspring health outcomes by mode of birth.

S4A Table. Complete case analysis of offspring health outcomes comparing planned repeat CS and unscheduled repeat CS with VBAC

| **Outcome** | **VBAC delivered offspring (reference category)** | | **Unscheduled repeat CS delivered offspring** | |  |  | **Planned repeat CS delivered offspring** | |  |  |
| --- | --- | --- | --- | --- | --- | --- | --- | --- | --- | --- |
|  |  |  | n outcome events/ total N | % | **Unadjusted risk of outcome** | **Adjusted risk of outcome** | n outcome events/ total N | % | **Unadjusted risk of outcome** | **Adjusted risk of outcome** |
| Obesity aged five years | 69/923 | 7.5 | 80/767 | 10.4 | OR **1.44 (1.03-2.02)** | OR 1.10 (0.77-1.56) †‡ | 245/1994 | 12.3 | **OR 1.73 (1.31-2.29)** | OR 1.11 (0.82-1.50) †‡ |
| Salbutamol inhaler use aged 5 years | 157/1862 | 8.4 | 152/1563 | 9.7 | OR 1.17 (0.93-1.48) | OR 1.15 (0.90-1.46) †* | 380/3887 | 9.8 | OR 1.18 (0.97-1.43) | OR 1.11 (0.90-1.37) †* |
| Hospitalisation with asthma | 203/7282 | 2.8 | 155/4944 | 3.1 | HR 1.16 (0.94-1.43) | HR 1.23 (0.99-1.51) †* | 336/10745 | 3.1 | HR 1.17 (0.99-1.40) | **HR 1.26 (1.04-1.52)** †* |
| Inflammatory bowel disease | 12/7282 | 0.2 | 7/4944 | 0.1 | HR 1.05 (0.41-2.67) | - | 4/10745 | 0.04 | HR 0.30 (0.10-0.94) |  |
| Type 1 diabetes mellitus | 27/7282 | 0.4 | 18/4944 | 0.4 | HR 1.03 (0.57-1.87) |  | 44/10745 | 0.4 | HR 1.20 (0.74-1.93) |  |
| Learning disability | 30/1679 | 1.8 | 29/1073 | 2.7 | OR 1.53 (0.91-2.56) | OR 1.37 (0.81-2.32) † | 31/1877 | 1.7 | OR 0.92 (0.56-1.53) |  |
| Cerebral palsy | 1/1679 | 0.06 | 2/1073 | 0.2 | OR 3.13 (0.28-34.65) |  | 2/1877 | 0.1 | OR 1.79 (0.16-19.74) |  |
| Cancer | 18/7282 | 0.2 | 11/4944 | 0.2 | HR 0.94 (0.44-1.99) |  | 24/10745 | 0.2 | HR 0.97 (0.53-1.79) |  |
| Death | 20/7282 | 0.3 | 11/4944 | 0.2 | HR 0.84 (0.40-1.75) |  | 20/10745 | 0.2 | HR 0.71 (0.38-1.32) |  |
| Death up to one year of age | 10/7282 | 0.1 | 3/4944 | 0.06 | HR 0.44 (0.12-1.61) |  | 3/10745 | 0.03 | HR **0.20 (0.06-0.74)** | HR 0.33 (0.08-1.32) † |

CS=cesarean section, VBAC=Vaginal birth after caesarean section, OR=odds ratio, HR=Hazard ratio, †adjusted for maternal age, gestation at birth, maternal Carstairs decile, maternal smoking status, birthweight, year of delivery, male infant, breastfeeding at six weeks, ‡adjusted for maternal BMI. *adjusted for maternal salbutamol prescription. Bold text indicates statistically significant findings at the 5% level. Blank cells indicate adjusted analyses not performed due to small number of events.

S4B Table. Complete case analysis of offspring health outcomes comparing planned repeat cesarean with unscheduled repeat cesarean delivery

| **Outcome** | **Unscheduled repeat CS delivered offspring (referent)** | | **Planned repeat CS delivered offspring** | |  |  |
| --- | --- | --- | --- | --- | --- | --- |
|  | n outcome events/ total N | % | n outcome events/ total N | % | **Unadjusted risk of outcome** | **Adjusted risk of outcome** |
| Obesity aged five years | 80/767 | 10.4 | 245/1994 | 12.3 | OR 1.20 (0.92-1.57) | OR 1.00 (0.75-1.33) †‡ |
| Salbutamol inhaler use aged 5 years | 152/1563 | 9.7 | 380/3887 | 9.8 | OR 1.01 (0.83-1.23) | OR 0.97 (0.80-1.20) †* |
| Hospitalisation with asthma | 155/4944 | 3.1 | 336/10745 | 3.1 | HR 1.01 (0.84-1.23) | HR 1.03 (0.84-1.26) †* |
| Inflammatory bowel disease | 7/4944 | 0.1 | 4/10745 | <0.1 | **HR 0.29 (0.09-0.99)** |  |
| Type 1 diabetes mellitus | 18/4944 | 0.4 | 44/10745 | 0.4 | HR 1.16 (0.67-2.01) |  |
| Learning disability | 29/1073 | 2.7 | 31/1877 | 1.7 | OR 0.61 (0.36-1.01) |  |
| Cerebral palsy | 2/1073 | 0.1 | 2/1877 | 0.1 | OR 0.57 (0.08-4.06) |  |
| Cancer | 11/4944 | 0.2 | 24/10745 | 0.2 | HR 1.02 (0.50-2.10) |  |
| Death | 11/4944 | 0.2 | 20/10745 | 0.2 | HR 0.84 (0.40-1.76) |  |

CS=cesarean section, VBAC=Vaginal birth after caesarean section, OR=odds ratio, HR=Hazard ratio,*adjusted for maternal salbutamol prescription, †adjusted for maternal age, gestation at birth, maternal Carstairs decile, maternal smoking status, birthweight, year of delivery, male infant, breastfeeding at six weeks, ‡adjusted for maternal BMI. Blank cells indicate adjusted analyses not performed due to small number of events.

S4C Table. Complete case analysis of offspring health outcomes comparing planned repeat cesarean with unscheduled births (unscheduled repeat CS and VBAC)

| **Outcome** | **Unscheduled births (referent)** | | **Planned repeat CS delivered offspring** | |  |  |
| --- | --- | --- | --- | --- | --- | --- |
|  | n outcome events/ total N | % | n outcome events/ total N | % | **Unadjusted risk of outcome** | **Adjusted risk of outcome** |
| Obesity aged five years | 149/1690 | 8.8 | 245/1994 | 12.3 | OR **1.45 (1.17-1.80)** | OR 1.05 (0.83-1.33) †‡ |
| Salbutamol inhaler use aged 5 years | 309/3425 | 9.0 | 380/3887 | 9.8 | OR 1.09 (0.93-1.28) | OR 1.04 (0.88-1.23) †* |
| Hospitalisation with asthma up to 30 months of age | 97/12226 | 0.8 | 95/10745 | 0.9 | HR 1.11 (0.84-1.48) | HR 1.19 (0.87-1.62) †* |
| Hospitalisation with asthma over 30 months of age | 261/12109 | 2.2 | 241/10641 | 2.3 | HR 1.15 (0.97-1.37) | HR 1.13 (0.94-1.37) †* |
| Inflammatory bowel disease | 19/12226 | 0.2 | 4/10745 | <0.1 | HR **0.30 (0.10-0.86)** |  |
| Type 1 diabetes mellitus | 45/12226 | 0.4 | 44/10745 | 0.4 | HR 1.18 (0.78-1.79) |  |
| Learning disability | 59/2752 | 2.1 | 31/1877 | 1.7 | OR 0.77 (0.49-1.19) | OR **0.59 (0.37-0.94)** †***** |
| Cerebral palsy | 3/2752 | 0.1 | 2/1877 | 0.1 | OR 0.98 (0.16-5.86) |  |
| Cancer | 29/12226 | 0.2 | 24/10745 | 0.2 | HR 0.99 (0.58-1.70) |  |
| Death | 31/12226 | 0.3 | 20/10745 | 0.2 | HR 0.76 (0.43-1.33) |  |

CS=cesarean section, VBAC=Vaginal birth after caesarean section, OR=odds ratio, HR=Hazard ratio,*adjusted for maternal salbutamol prescription, †adjusted for maternal age, gestation at birth, maternal Carstairs decile, maternal smoking status, birthweight, year of delivery, male infant, breastfeeding at six weeks, ‡adjusted for maternal BMI. Blank cells indicate adjusted analyses not performed due to small number of events.
