# Supplementary material for: Planned Repeat Cesarean Section at Term and Adverse Childhood Health Outcomes: A Record-Linkage Study
Source: PLoS Med. 2016 Mar 15;13(3):e1001973. doi: 10.1371/journal.pmed.1001973 (PMC4792387; doi:10.1371/journal.pmed.1001973)
Supplement: S2 Text — (DOCX) [file pmed.1001973.s006.docx]

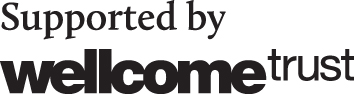


**Research Protocol**

**Wellcome Trust Research Training Fellowship**

**Offspring risks of elective caesarean (OREC)**

**Ref no. 2/038/13**

**Fellow; Dr Mairead Black, Clinical lecturer, Obstetrics and Gynaecology**

**Supervisors; Professor Siladitya Bhattacharya, Dr David Mclernon**

Version 3.2 26/8/2012

**Contents**

Study team contact details 1

Abstract 2

Lay summary 2

Background 3

Pilot work 3

Aims 4

Research questions 4

Methods 4

Data handling 7

Ethics and NHS R&D approvals 7

Permissions to use data 7

Risks/study weaknesses 7

Costings 7

Proposed timeline 7

Project management 7

Potential future work 7

Potential benefit of research to the NHS/patients/public health 8

References 8

**Study Team Contact Details**

Dr Mairead Black

School of Medicine and Dentistry
Division of Applied Health Sciences
University of Aberdeen
Room 3 Dugald Baird Centre for Research on Women's Health
Aberdeen Maternity Hospital
Cornhill Road, Aberdeen AB25 2ZD
Tel:  +44(0)1224 438420
Fax:  +44(0)1224 438486
e-mail: [mairead.black@abdn.ac.uk](mailto:mairead.black@abdn.ac.uk)

Dr David Mclernon

Division of Applied Health Sciences

University of Aberdeen

Medical Statistics Team

Room 1:028, Polwarth Building
Foresterhill
Aberdeen AB25 2ZD
Tel +44 (0) 1224 437152
Fax +44 (0) 1224 437285
email [d.mclernon@abdn.ac.uk](mailto:d.mclernon@abdn.ac.uk)

Professor Siladitya Bhattacharya

School of Medicine and Dentistry

Division of Applied Health Sciences

University of Aberdeen

Aberdeen Maternity Hospital

Foresterhill, Aberdeen AB25 2ZD

Tel: +44 1224 438419

Fax: +44 1224 438486

**Abstract**

One in four babies in the UK are delivered by caesarean section. A significant proportion of these deliveries are not considered necessary given the associated profile of risks and benefits to the woman and offspring. In order to make an informed choice between planned caesarean section and attempting vaginal delivery, women and healthcare providers require as much information as possible on the associated risks and benefits. Currently, the published literature lacks population-based data on the medium or long-term implications for offspring delivered by planned caesarean section. There are a small number of studies which suggest that asthma, obesity, inflammatory bowel disease, type-1 diabetes and cancer of various types may be more common following any caesarean delivery, but these have not necessarily considered whether planned caesarean delivery also carries these risks. In addition, the risk of special educational needs has been described as being increased when children are born as little as 1-2 weeks before their due date. This is relevant to planned caesarean birth, as such deliveries are routinely performed one week before the due date. Death in childhood following delivery by planned caesarean section has not been studied.

In this population-based retrospective cohort study, data from various sources held by Information Services Division Scotland will be used to assess the risk of asthma, obesity, type-1 diabetes, inflammatory bowel disease, cancer, special educational needs, cerebral palsy and death in offspring up to the age of 18 delivered by planned caesarean section. The comparison group will comprise all equivalent offspring delivered vaginally or by emergency caesarean section. The primary analysis will compare these outcomes in first offspring (cohort 1), while a second analysis will compare these outcomes in offspring where the mother previously delivered a child by caesarean section (cohort 2). This second comparison will ensure that the findings are relevant to women faced with time to consider her options of repeat CS or attempted vaginal birth. This is a common scenario and is the single clinical situation where providing more information on risks and benefits of mode of delivery options has the most potential to alter the overall caesarean section rate. The risk estimates will be presented as odds ratios, which will be calculated using logistic regression models adjusting for known potential confounding factors. The results will be directly applicable to women who are faced with a choice between planned caesarean birth and attempted vaginal delivery.

**Lay summary**

One in four women deliver their babies by caesarean section (CS), with many of these being performed at the request of the woman and partner in the absence of absolute medical indications. The long-term risks to the offspring of being deprived of exposure to labour are poorly understood, but may involve impaired intellectual development, impaired development of the immune system and increased risk of obesity. The proposed study will use Scottish data to investigate whether planned CS increases the risk of certain childhood health conditions compared with planning vaginal birth. Ultimately, this study aims to gather information which could contribute to optimising CS rates in the future.

**Background**

The current UK caesarean section (CS) rate is at a record high, with at least one in four babies delivered this way (National Institute for Health and Clinical Excellence 2011). As one of the commonest reasons cited for performing CS is a history of previous CS in the mother, there is real potential for influencing the overall CS rates if the evidence base regarding vaginal birth after CS was expanded. In addition, CS at maternal request where no medical indication exists is a growing phenomenon. In order to ensure that such deliveries follow fully informed consent, more work is needed to explore the consequences of this mode of birth for offspring(National Institute for Health and Clinical Excellence 2011). The associated risks to offspring of avoiding labour and delivery may include impaired immunity(Strachan 2000) and inhibited stress response(Taylor, Fisk & Glover 2000). The potential consequences of impaired immune development include an increased risk of asthma(Magnus et al. 2011), type 1 diabetes(Cardwell et al. 2008) and potentially inflammatory bowel disease and childhood cancer. Associations between delivery by CS and childhood obesity have also been recognised(Goldani et al. 2011). As planned CS delivery is almost always performed at least one week before the due date, the risk of shortened gestation on special educational needs with or without cerebral palsy has been identified(MacKay et al. 2010). It is not clear whether these various associations may be explained by lack of exposure to labour following prelabour CS, or simply avoidance of vaginal delivery, as with CS performed at any time. In addition, it is not known whether risk of death in childhood is in any way associated with mode of delivery. If so, this would prompt further study to investigate whether there are currently unrecognised confounding factors which explain such a relationship, or whether the association persists. As studies performed to date have looked at offspring outcomes following delivery by CS including both planned and emergency procedures, this study intends to explore the risks associated specifically with planned CS delivery. Such an approach allows the results to be generalisable to couples who are taking time to consider the risks and benefits of a planned CS delivery of their offspring. As the risk of such conditions following emergency CS delivery would never be expected to take priority over the indication for emergency CS, making the consequences of planned CS the main focus of this study is considered a pragmatic approach to yielding results which are highly applicable in clinical practice.

**Pilot work**

My MSc project titled ‘Mode of delivery after caesarean section; costs and consequences’ has provided pilot data to inform sample size calculation. Data collected demonstrates that 3.6% of all women delivering each year in Aberdeen are women in their second pregnancy with a history of previous CS, and that of these, 60% will attempt VBAC. This enabled a sample size calculation for the second cohort included in this study (those women who delivered a first child by caesarean section).

**Aims**

The aims of this project are to identify any associations between planned caesarean section and development of immune-related childhood conditions, obesity, additional educational support needs and death.

**Research Questions**

1. Compared with first offspring delivered following a plan for vaginal birth, do offspring of the first pregnancy delivered by planned CS have an increased risk of childhood i) asthma-related illness ii) asthma requiring hospital admission iii) type 1 diabetes iv) inflammatory bowel disease v) obesity in primary one vi) additional educational support needs vii) cerebral palsy viii) childhood cancer ix) death?
2. Compared with offspring delivered following a plan for vaginal birth after previous CS, do offspring delivered by planned repeat CS have an increased risk of childhood i) asthma-related illness ii) asthma requiring hospital admission iii) type 1 diabetes iv) inflammatory bowel disease v) obesity in primary one vi) additional educational support needs vii) cerebral palsy viii) childhood cancer ix) death?

**Methods**

Study design; Retrospective cohort study

Exposure; Delivery by planned caesarean section

Outcomes; Childhood i) asthma-related illness ii) asthma requiring hospital admission iii) type 1 diabetes iv) inflammatory bowel disease v) obesity in primary one vi) additional educational support needs vii) cerebral palsy viii) cancer ix) death.

Study population and period;

Cohort 1; All offspring of women delivering singleton infants in their first pregnancy between 1993 and 2007.

Cohort 2; All offspring of women delivering a second infant between 1993 and 2007 after a caesarean section in their first pregnancy.

Inclusion criteria; Singleton offspring, livebirths, twenty four completed weeks gestation at delivery.

Exclusion criteria; Stillbirths and miscarriages, multiple pregnancy.

Data sources; All via information services division (ISD) Scotland. Details of these are listed in table 1.

Table 1. Data Sources;

|  | Source (all obtained via ISD) | Completeness | Dates available | Quality | Notes |
| --- | --- | --- | --- | --- | --- |
| Study Population | SMR02 | 99.9% | 1993-2007+ |  |  |
| Asthma-related illness | PIS | 99% (salbutamol inhaler) | 2009+ |  | Will include those with wheeze alone |
| Asthma requiring hospital admission | SMR01 |  | 1993-2007+ |  |  |
| Type 1 Diabetes | PIS | 99% (insulin prescription) | 2009+ |  |  |
| Inflammatory bowel disease | SMR01 |  | All |  | Will miss cases not admitted |
| Obesity | CHSS | 74% | 2009+ |  | Recorded in primary one |
| Additional educational support needs | SNS | Missing; certain regions, particulary historically. Those without consent. | Varies by region |  |  |
| Cerebral palsy | SNS | Missing; certain regions, particulary historically. Those without consent. | Varies by region |  |  |
| Cancer | Scottish cancer registry | 100% |  | 97% accurate |  |
| Death | NSS (via NRS) | 99% |  |  |  |
| Infant feeding method at 6-8 weeks of age | CHSP Pre-school |  | 2010 (all Scotland)  2005 – approximately half of Scotland |  |  |

Asthma – Source 1; Acute hospital discharge information (SMR01) with linkage to mothers possible using children’s CHI numbers, Source 2; Prescription information system (any prescription for ventolin inhaler) with linkage to mothers made possible using the children’s CHI number (second source provides data on all asthma-related illness, while first source includes only those cases of asthma severe enough to warrant hospital admission).

Type 1 diabetes – Source 1; Prescription information system (any prescription for insulin) with linkage to mothers made possible using the children’s CHI number, Source 2; SCI database with linkage to mothers made possible using the children’s CHI number (second source provides quality control check on data and potentially highlights missing cases from source 1 and date of diagnosis).

Inflammatory bowel disease – acute hospital discharge information (SMR01) on any cases of Crohn’s or ulcerative colitis.

Special educational needs and cerebral palsy – Support needs system (Scotland) with linkage to mothers made possible using the children’s CHI numbers.

Obesity – Child health surveillance programme (Scotland) primary 1 review data, with linkage to mothers made possible using the children’s CHI numbers.

Cancer – Scottish cancer registry accessed via ISD

Death – Data on death in childhood is help in NSS but accessed via NRS when part of a data-linkage study.

Infant feeding method – Pre-school child health surveillance programme record of method of infant feeding at 6-8 weeks of age.

Statistical analysis; Outcomes will be assessed within cohort 1 and cohort 2 separately. Odds ratios will be used to measure the risk of developing the conditions in question following delivery by planned CS. Pilot data suggests an estimated sample size of 53,000 exposed (42,000 cohort 1 and 11,000 cohort 2) and 419,000 unexposed (401,000 cohort 1 and 17,000 cohort 2)^20^. A power calculation found that a two group continuity corrected chi-squared test with a 0.05 two-sided significance level will have 99% power to detect the difference between the proportion of childhood asthma in the exposed group (cohorts 1 and 2) of 0.12 and the unexposed groups (cohorts 1 and 2) of 0.14 (odds ratio of 1.194) when the sample sizes are as outlined above. Asthma was chosen as the primary outcome due to its relatively high prevalence (20-30%) in the UK{{697 Anderson,H.R. 2007}}.

Analysis: Multivariable logistic regression models will be used to calculate the odds ratio of each of the outcomes of interest for ECS versus emergency CS or vaginal birth. The models will be adjusted for known confounders identified on univariate analysis that were significantly different between the two groups. Potential confounders to be tested are presented according to outcome of interest in table 2.

Table 2. Potential confounding factors for each of the outcomes of interest.

| **Outcome** | **Potential confounders** |
| --- | --- |
| Asthma-related illness | Maternal age  Gender of offspring  Maternal social class  Maternal smoking status*  Standardised birthweight  Gestation at delivery  Breast feeding |
| Asthma requiring hospital admission | Maternal age  Gender of offspring  Maternal social class  Maternal smoking status*  Standardised birthweight  Gestation at delivery  Breast feeding |
| Type 1 diabetes | Maternal history of type 1 diabetes |
| Inflammatory bowel disease |  |
| Obesity | Maternal weight  Maternal BMI (where available)  Maternal social class |
| Additional educational support needs | Standardised birthweight  Gestation at delivery  Maternal social class |
| Cerebral palsy | Standardised birthweight  Gestation at delivery  Maternal social class |
| Death | Maternal social class  Maternal smoking status* |

*may not meet true confounder criteria as not necessarily associated with mode of delivery except perhaps via social class route

**may not meet true confounder criteria as not necessarily associated with mode of delivery except on the causal pathway

Year of delivery will be included as a covariate for all outcomes to account for changes in data coding and medical practice over time.

**Data Collection/handling**

A safehaven (DASH) will be utilised to enable linkage of data using the CHI numbers of mothers and offspring. This will ensure that I as the researcher will not handle potentially identifiable data.

**Ethics and NHS R&D approvals**

Ethical approval will be sought from the North of Scotland Research Ethics Committee. Version 2 of the protocol has been reviewed by NHS R&D and R&D approval is not deemed necessary.

**Permissions to use data**

Approval will be sought from the ISD privacy advisory committee (PAC).

**Risks/Study Weaknesses**

Lack of ability to adjust for potential confounding effect of maternal BMI, breast-feeding status or family history of asthma or inflammatory bowel disease.

**Costings**

The funding provided by Wellcome Trust will cover data extraction from ISD, data management by UoA DaSH and purchase of computer storage device to a total of £7350.

**Proposed timeline**

With data requested during spring 2013, data is expected to be received during autumn 2013, with analysis complete by early 2014.

**Project management**

The research fellow will be responsible for project management and will be supervised by both Professor Bhattacharya and Dr David Mclernon.

**Potential future work**

This project is part of a research training fellowship which has two further components which together will provide tools of use during future development of complex interventions aimed at optimising the CS rate.

**Potential benefit of research to the NHS/patients/public health**

The long-term consequences of CS are of great interest to women considering their options when faced with a decision regarding how to attempt delivery of a child, an option commonly given to couples following one previous CS. Adding such information to that which is currently considered during the decision-making process is likely to influence the ultimate CS rate, and provide greater patient satisfaction. If greater risks of chronic health problems are recognised when labour is avoided, measures to increase attempts to labour could ultimately reduce the global burden of chronic disease along with morbidity of multiple repeat CS. There is also potential for such information to influence women’s choices across the world where CS for no medical indication is a rapidly growing phenomenon. In order to maximise external validity of the study, we are considering the risks of the health conditions in question among both first-born children and second-born children where the previous child was delivered by CS.

**References**

Cardwell, C.R., Stene, L.C., Joner, G., Cinek, O., Svensson, J., Goldacre, M.J., Parslow, R.C., Pozzilli, P., Brigis, G., Stoyanov, D., Urbonaite, B., Sipetic, S., Schober, E., Ionescu-Tirgoviste, C., Devoti, G., de Beaufort, C.E., Buschard, K. & Patterson, C.C. 2008, "Caesarean section is associated with an increased risk of childhood-onset type 1 diabetes mellitus: a meta-analysis of observational studies", *Diabetologia,* vol. 51, no. 5, pp. 726-735.

Goldani, H.A., Bettiol, H., Barbieri, M.A., Silva, A.A., Agranonik, M., Morais, M.B. & Goldani, M.Z. 2011, "Cesarean delivery is associated with an increased risk of obesity in adulthood in a Brazilian birth cohort study", *The American Journal of Clinical Nutrition,* vol. 93, no. 6, pp. 1344-1347.

MacKay, D.F., Smith, G.C., Dobbie, R. & Pell, J.P. 2010, "Gestational age at delivery and special educational need: retrospective cohort study of 407,503 schoolchildren", *PLoS medicine,* vol. 7, no. 6, pp. e1000289.

Magnus, M.C., Haberg, S.E., Stigum, H., Nafstad, P., London, S.J., Vangen, S. & Nystad, W. 2011, "Delivery by Cesarean section and early childhood respiratory symptoms and disorders: the Norwegian mother and child cohort study", *American Journal of Epidemiology,* vol. 174, no. 11, pp. 1275-1285.

National Institute for Health and Clinical Excellence 2011, *Caesarean Section; Clinical Guideline No. 132*, NICE.

Strachan, D.P. 2000, "Family size, infection and atopy: the first decade of the "hygiene hypothesis"", *Thorax,* vol. 55 Suppl 1, pp. S2-10.

Taylor, A., Fisk, N.M. & Glover, V. 2000, "Mode of delivery and subsequent stress response", *Lancet,* vol. 355, no. 9198, pp. 120.
